# Patient Factors Associated with Interest in Teledermatology: Cross-sectional Survey

**DOI:** 10.2196/21555

**Published:** 2021-05-10

**Authors:** Maham Ghani, Colin Adler, Howa Yeung

**Affiliations:** 1 City University of New York School of Medicine City University of New York New york, NY United States; 2 Department of Dermatology, Emory University School of Medicine Emory University Atlanta, GA United States; 3 Regional Telehealth Service, Veterans Affairs Veterans Integrated Service Network 7 Decatur, GA United States

**Keywords:** digital divide, e-health literacy, Internet, modifiable behaviors, teledermatology

## Abstract

**Background:**

Teledermatology is a conduit for patients communicating with dermatologists on the internet, which bypasses in-person visits. It holds promise to address access needs for dermatologic care; however, the interest in using teledermatology is unknown in underserved populations with potential barriers to the use of health care technology.

**Objective:**

This study aimed to characterize the association between demographic characteristics with interest in exchanging digital images or videos of skin lesions with health care providers electronically.

**Methods:**

We examined data from the Health Information National Trends Survey (HINTS) 4 cycle 4 (2014) of the National Cancer Institute. HINTS is a cross-sectional, nationally representative household survey conducted annually, which collects information on demographics, perceptions and use of health information, and provides information on how cancer risks are perceived. HINTS 4 cycle 4 had a sample of 3677 participants. We examined the outcome to the question, “how interested are you in exchanging digital images or videos (eg, photos of skin lesions) with a health care provider electronically?” We dichotomized the outcome by a high level of interest (responding with “very”) and those who did not have a high level of interest (responding with “somewhat,” “a little,” or “not at all”) in exchanging images or videos. We used a multivariable logistic regression model developed through backwards selection, with all final covariates associated with varying levels of teledermatology use at *P*<.05. Sensitivity analysis was performed by changing the outcome dichotomy to model those who were “not at all” interested. Two-sided tests were performed with *P*<.05 considered significant.

**Results:**

Among 3447 respondents, 888 (weighted prevalence=26.2%) were “very” interested in participating in teledermatology. A higher interest in using teledermatology was associated with a younger age, higher educational attainment, higher household income, internet usage, type of mobile device ownership, history of electronic medical information exchange with a clinician within the past 12 months, and high level of trust in web-based information on cancer (for all, *P*<.01), but not with the female gender, race or ethnicity, health insurance status, or having a regular medical provider.

**Conclusions:**

Modifiable access barriers to teledermatology adoption include trust, experience with teledermatology, and use of health apps. Teledermatology program implementation should address these specific factors within the digital divide to promote equitable access to care across diverse patient populations.

## Introduction

Teledermatology is a conduit for patients communicating with dermatologists on the internet, which bypasses in-person visits. It can occur through a live videoconferencing or by sending photographs for asynchronous review [1]. Patients may work with their general practitioners (GP) to contact a dermatologist via telehealth, or they can initiate the interaction themselves directly [2]. With evolving technology, camera phones can click photographs of sufficient quality to meet teledermatology standards, with potential for broad patient adoption without facilitation from an intermediary GP [2]. Implementation of teledermatology has recently been accelerated during the 2020 COVID-19 pandemic [3].

Teledermatology has the ability to increase medical care access to diverse populations at reduced costs and wait times [1,4]. High levels of concordance in diagnosis have been noted between teledermatology and in-person consultations [5]. However, there remains a knowledge gap in the characteristics of patients who use teledermatology. Previous studies suggest that demographics such as young age, high income, and high educational status are correlated with increased eHealth literacy, which refers to the ability to search, obtain, and understand web-based health information [6], as well as increased health app usage [7] and communication with physicians on the internet [8]. However, these studies did not explore the factors that influence patient interest in participating specifically in teledermatology.

Consideration of the identified characteristics of individuals who are less interested in using teledermatology in the context of the digital divide has implications for health equity as teledermatology expands. The digital divide encompasses a broad range of variables that contribute to the gap in the ability to access and use digital devices [9]. The exchange of images or videos distinguishes teledermatology from the more general telemedicine. Exchanging images requires more advanced technologic skills, with particular attention to be paid to aspects pertaining to image quality such as focus, lighting, and background [10]. Teledermatology requires a minimal bandwidth to adequately participate in videoconferencing [10]. Identifying the images of the patient may pose a potential vulnerability and require substantial patient trust in the technological platform.

In this study, we aimed to determine sociodemographic correlates with patient interest in exchanging images and videos electronically with health care providers in the nationally representative Health Information National Trends Survey (HINTS) of the National Cancer Institute. We hypothesized that teledermatology adoption by various subpopulations may be mediated by differential levels of access to and interest in exchanging images and videos with their health care providers.

## Methods

### Study Sample

We examined data from HINTS 4 cycle 4 (2014) of the National Cancer Institute. HINTS is a cross-sectional, nationally representative household survey conducted annually, which collects information on demographics and the perceptions and use of health information, and provides information on how cancer risks are perceived. HINTS 4 cycle 4 had a sample of 3677 participants. Data from 2014 were used because the outcome of interest, “How interested are you in exchanging digital images or video (eg, photos of skin lesions) with a health care provider electronically?” was only available in this cycle. Details about HINTS data collection, including weighting methodologies, is described elsewhere. The institutional review board of Emory University exempted this study from review.

### Sociodemographic and Health Behavior Correlates

The covariates include sociodemographic variables as well as access and use of the internet such as the following: self-reported gender, age, education, race and ethnicity, annual household income level, seeing a health professional regularly, having health insurance, using the internet, using electronic devices to share medical information with a health professional, and using various devices (if any) with or without health apps on them. The survey question on devices was, “please indicate if you have one of the following electronic devices: tablet, smartphone, cellphone, etc.” The responses were as follows: “(1) tablet computer like an iPad, Samsung, Galaxy Tab, Motorola Xoom, or Kindle Fire, only; (2) smartphone such as an iPhone, Android phone, Blackberry device, or a Windows phone; (3) basic cellphone only; and (4) multiple devices listed.” The survey question regarding health apps contained on one’s electronic device was, “on your tablet or smartphone, do you have any software apps related to health?” The answer choices were as follows: “yes,” “no,” and “don’t know.” These 2 questions were combined to generate 1 variable to specify the type of electronic device a participant owned and if there were health apps installed on that device.

We also assessed cancer information–seeking behavior, including, but not limited to, skin cancer. Cancer-related covariates included the following: seeking cancer information, trusting web-based information on cancer, and using the internet to obtain cancer-related information for oneself in the past 12 months.

### Outcome: Interest in Using Teledermatology

The primary outcome was defined by the following question: “how interested are you in exchanging information like digital images or video (eg, photos of skin lesions) with a health care provider electronically?” We dichotomized the outcome by a high level of interest (responding with “very”) and those who did not have a high level of interest (responding with “somewhat,” “a little,” or “not at all”) in exchanging images and videos with health care providers.

### Statistical Analysis

Statistical analyses were conducted using SAS (version 9.4, SAS Institute). Nationally representative prevalence estimates were obtained using jackknife replicate weights that accounted for the complex survey design. For bivariate analyses, the associations between specific sociodemographic groups and interest in sharing photographs or videos were assessed using unconditional logistic regression. We used complete cases analysis for logistic regression owing to the low proportion of missing data (6%). A multivariable logistic regression model was developed through backwards selection, with all final covariates associated with varying levels of teledermatology use at *P*<.05. Furthermore, sensitivity analysis was performed by changing the outcome dichotomy to model those who were “not at all” interested. Two-sided tests were performed with *P*<.05 indicating statistical significance.

## Results

The response rate of HINTS 4 cycle 4 was 34%. In total, 3677 respondents fully completed 3529 surveys and partially completed 148 surveys. Demographic characteristics of survey respondents by the level of their interest in teledermatology are summarized in Table 1. A total of 888 of 3447 (weighted prevalence=26.2%) participants were very interested, 784 (22.8%) were somewhat interested, 515 (16.2%) were a little interested, and 1260 (34.8%) were not at all interested.

As shown in Table 1, the socioeconomic demographic characteristics associated with high levels of interest in sharing photographs or videos were the female gender (*P*=.02), young age (*P*<.001), high levels of education (*P*=.001), high annual household income range (*P*<.001), having a regular medical provider (*P*=.02), trusting web-based information on cancer (*P*<.001), using the internet (*P*<.001), sharing medical information with medical providers (*P*<.001), and having multiple electronic devices, including smartphones and tablets, with health apps (for both *P*<.001).

Multivariable modeling in Table 2 shows that trust in web-based information on cancer (odds ratio [OR] 1.9, 95% CI 1.3-2.8) is associated with high levels of interest in exchanging images.

Sharing medical information electronically with a health care professional (OR 2.1, 95% CI 1.5-2.9) is associated with high levels of interest in exchanging images (Table 2). Sensitivity analysis revealed that individuals who reported no interest in exchanging images were less likely to have shared medical information electronically with a health care professional (OR 0.3, 95% CI 0.2-0.4).

Having multiple devices with health apps (OR 2.6, 95% CI 1.5-4.6) is associated with high levels of interest in exchanging images (Table 2). Sensitivity analysis revealed that individuals reporting no interest in exchanging images were less likely to own multiple devices with health apps (OR 0.3, 95% CI 0.2-0.5), own multiple devices without health apps (OR 0.5, 95% CI 0.3-0.7), own a tablet device without health apps (OR 0.6, 95% CI 0.4-0.9), own a smartphone with health apps (OR 0.4, 95% CI 0.2-0.8), and own a smartphone without health apps (OR 0.5, 95% CI 0.3-0.7).

Age, gender, annual household income, education, having a regular medical provider, internet usage, having health insurance, and trusting web-based information on cancer were not significant predictors of a high level of interest in teledermatology after adjusting for the aforementioned variables.

**Table 1:** Patient factors associated with high levels of interest in exchanging digital images (eg, photographs of skin lesions) with a health care provider electronically.

Factors			Very interested (n=888), n (weighted %)		Not at all interested (n=2559), n (weighted %)		Total participants		*P* value	
**Gender**										.02
	Male	322 (25.1^a^)		1018 (74.9)		1340				
	Female	557 (27.7)		1495 (72.3)		2052				
	Unknown	9 (9.2)		46 (90.8)		55				
**Age** **(years)**										<.001
	18-34	153 (30.0)		310 (70.0)		463				
	35-49	243 (32.2)		474 (67.8)		717				
	50-64	295 (22.3)		876 (77.7)		1171				
	≥65	166 (16.1)		780 (83.9)		946				
	Unknown	31 (19.0)		119 (81.0)		150				
**Education**										.001
	High school or below	56 (19.0)		200 (81.0)		256				
	High School	126 (21.7)		479 (78.3)		605				
	Some college degree	256 (24.4)		788 (75.6)		1044				
	College or higher	424 (31.7)		994 (68.3)		1418				
	Unknown	26 (18.8)		98 (81.2)		124				
**Race and ethnicity**										.61
	Hispanic	143 (29.3)		343 (70.7)		486				
	Non-Hispanic White	445 (24.6)		1447 (75.4)		1892				
	Non-Hispanic Black	152 (30.4)		361 (69.9)		513				
	Other	63 (28.8)		166 (71.2)		229				
	Unknown	85 (25.9)		242 (74.1)		327				
**Income range**										<.001
	<US $20,000	157 (24.8)		537 (75.2)		694				
	US $20,000-$34,999	109 (22.2)		348 (77.8)		457				
	US $35,000-$49,999	118 (24.2)		347 (75.8)		465				
	US $50,000-$74,999	132 (26.5)		396 (73.5)		528				
	≥US $75,000	321 (33.0)		643 (67.0)		964				
	Unknown	51 (11.1)		288 (88.9)		339				
**Having a regular medical provider**										.02
	Yes	627 (27.7)		1783 (72.3)		2410				
	No	260 (23.7)		760 (76.3)		1020				
	Unknown	1 (2.1)		16 (97.9)		17				
**Having health insurance**										.15
	Yes	773 (25.7)		2263 (74.3)		3036				
	No	113 (29.7)		284 (70.3)		397				
	Unknown	2 (9.7)		12 (90.3)		14				
**Trust in web-based information on cancer**										<.001
	A lot	231 (38.4)		407 (61.6)		638				
	Not a lot	620 (23.8)		1997 (76.2)		2617				
	Unknown	37 (20.8)		155 (79.2)		192				
**Using the internet**										<.001
	Yes	767 (27.5)		1977 (72.5)		2744				
	No	117 (17.8)		580 (82.2)		697				
	Unknown	4 (85.7)		2 (14.3)		6				
**Owning electronic devices and using health apps**										<.001
	Multiple devices with health apps	220 (40.9)		292 (59.1)		512				
	Multiple devices without health apps	187 (25.0)		508 (75.0)		693				
	Tablet device with health apps	14 (30.6)		51 (69.4)		65				
	Tablet device without health apps	68 (25.4)		213 (74.6)		281				
	Smartphone with health apps	73 (34.4)		150 (65.6)		223				
	Smartphone without health apps	142 (23.4)		421 (76.6)		563				
	Basic cellphone	117 (15.2)		631 (84.8)		748				
	None	42 (11.0)		249 (89.0)		291				
	Unknown	25 (43.7)		44 (56.3)		69				
**Sharing medical information with health care providers**										<.001
	Yes	371 (39.6)		572 (60.4)		943				
	No	507 (20.9)		1954 (79.1)		2461				
	Unknown	10 (13.9)		33 (86.1)		43				

^a^Weighted percentage to adjust for the nonresponse bias.

**Table 2:** Multivariable analysis of interest levels in exchanging digital images (eg, photographs of skin lesions) with a health care provider electronically.

Characteristics		Very interested			Not at all interested	
		Odds Ratio (95% CI)	*P* value	Odds ratio (95% CI)		*P* value
**Gender**		N/A^a^	.51			.20
	Male	Ref^b^		Ref		
	Female	1.1 (0.8-1.7)		1.2 (0.9-1.6)		
**Age (years)**			.06			.10
	18-34	Ref		Ref		
	35-49	1.2 (0.8-1.9)		0.9 (0.7-1.4)		
	50-64	0.8 (0.5-1.2)		1.3 (0.9-1.8)		
	≥65	0.7 (0.4-1.2)		1.5 (1.0-2.1)		
**Trusting web-based information on cancer**			.002			.80
	Yes	1.9 (1.3-2.8)		0.9 (0.7-1.3)		
	No	Ref		Ref		
**Sharing medical information with health care providers**			<.001			<.001
	Yes	2.1 (1.5-2.9)		0.3 (0.2-0.4)		
	No	Ref		Ref		
**Owning devices with or without health apps**			<.001			<.001
	Multiple devices with health apps	2.6 (1.5-4.6)		0.3 (0.2-0.5)		
	Multiple devices without health apps	1.5 (0.8-2.7)		0.5 (0.3-0.7)		
	Tablet device with health apps	1.7 (0.6-4.5)		0.9 (0.3-2.6)		
	Tablet device without health apps	1.6 (0.8-3.3)		0.6 (0.4-0.9)		
	Smartphone with health apps	2.0 (0.9-4.0)		0.4 (0.2-0.8)		
	Smartphone without health apps	1.3 (0.7-2.4)		0.5 (0.3-0.7)		
	Basic cellphone	Ref		Ref		
	None	0.7 (0.3-1.4)		1.5 (1.0-2.1)		

^a^N/A: not applicable.

^b^Ref: Reference group for comparison.

## Discussion

### Principal Findings

High levels of interest in using teledermatology were associated with modifiable behaviors such as the use of devices with health apps, trust in web-based information on cancer, and prior experiences in exchanging health information with physicians on the internet. Sociodemographic factors such as young age, female gender, high education, and high household income were not associated with an increased interest in exchanging images of skin lesions with health care providers after adjusting for these modifiable variables. Future implementation of teledermatology should address these identified factors within the digital divide to provide equitable access to care across diverse patient populations.

Access to devices and how they are used are aspects of the digital divide, which can be adjusted. Physical access to the internet was found to be the most significant predictor of web-based patient-provider communication [9]. Once access is established, usage becomes the rate-limiting factor, which depends on the ability to retrieve and search for information on the internet and to use mobile health apps [11,12]. Users of mobile health apps are more likely to exhibit health-promoting behaviors than those who own similar devices but do not use health apps [11]. A potential barrier to the use of health apps is privacy concerns with inputting personal data into digital devices. Digital health information requires high levels of eHealth literacy to effect action [13]. Mobile device and health app usage is associated with characteristics previously linked to increased eHealth literacy, such as young age, higher education, and high income [7,14]. However, unlike age, education, and income, access and usage of devices can be modified to enhance teledermatology implementation.

Trust in web-based information on cancer can be directed to mediate interest in teledermatology usage. Trust is a necessary antecedent to the development of eHealth literacy and engagement with health information [15,16]. Trust in web-based health information is associated with higher education and the disclosure of health information on the internet, which are factors linked to eHealth literacy [17]. Even among groups with increased eHealth literacy, young individuals have higher trust in web-based health care services than their older counterparts [18]. Patient trust in a telemedicine service can be broken down into their trust in the organization, treatment, care professional, and technology [19]. Increased trust in the telehealth service can be gained when patients are referred by other health care professionals [20]. Moreover, face-to-face interactions with the provider prior to a web-based consultation also increases patient trust [21]. The provider should ensure that patient concerns are being addressed as this will increase trust in the telehealth provider and the treatment plan [21].

Prior experience in exchanging health information on the internet is an adjustable factor that can be targeted to increase interest in exchanging digital images. Lack of knowledge and experience with web-based patient-provider communication is found to impede its use [9], while prior experience with sharing medical information electronically is associated with higher interest in exchanging digital images with providers [15]. As patients expand their experiences with digital technology within and outside of the health care context, patients will have the opportunity to develop trust in teledermatology services [19].

Teledermatology interventions are implemented and expanded across populations to bridge the digital divide [22]. For example, the US Department of Veteran Affairs has expanded the reach of teledermatology by loaning electronic devices to veterans and provided training in using the devices so that veterans can more easily connect with the existing telehealth networks [23]. They have also attempted to evaluate the effectiveness of these measures through a survey on patient satisfaction with teledermatology use and its contributory factors [24]. Our study found that factors malleable to influence—use of health apps, trust, and experience—are barriers that can be mediated to increase the reach, adoption, and effectiveness of teledermatology.

### Limitations

There were some limitations to this study. The cross-sectional nature of the HINTS data precluded the establishment of causal relationships between the usage, trust, and prior experience in using health apps and the interest in teledermatology. The measured outcome was available only in HINTS 4 cycle 4 (2014); more recent data were not available to address the study question. All survey responses were self-reported and subject to information bias. We could not exclude residual confounding variables from additional unmeasured or unexamined variables. We were unable to distinguish the history of skin cancer from that of other cancers when controlling for covariates related to information-seeking behaviors associated with cancer. Interest in exchanging images on the internet might differ if the patient worked with GPs to send images to a teledermatology service or if the service is directly patient-initiated, and this aspect should be examined in future studies. Future studies should explore how well patients and GPs follow teledermatology guidelines on taking adequate images. We were unable to assess if interests in teledermatology translated directly to teledermatology usage or adherence to recommendations from teledermatology services.

### Conclusions

In conclusion, modifiable access barriers to teledermatology adoption included experience with exchanging health information on the internet, trust in web-based information on cancer, and the use of mobile health apps. Future implementation of teledermatology should address these identified factors within the digital divide to provide equitable access to care across diverse patient populations.

## Abbreviations

GP: general practitioner
HINTS: Health Information National Trends Survey

## References

[1] Coates SJ, Kvedar J, Granstein RD (2015). Teledermatology: from historical perspective to emerging techniques of the modern era: part I: History, rationale, and current practice. J Am Acad Dermatol.

[2] Walocko FM, Tejasvi T (2017). Teledermatology Applications in Skin Cancer Diagnosis. Dermatol Clin.

[3] Hollander JE, Carr BG (2020). Virtually Perfect? Telemedicine for Covid-19. N Engl J Med.

[4] Uscher-Pines L, Malsberger R, Burgette L, Mulcahy A, Mehrotra A (2016). Effect of Teledermatology on Access to Dermatology Care Among Medicaid Enrollees. JAMA Dermatol.

[5] Bashshur RL, Shannon GW, Tejasvi T, Kvedar JC, Gates M (2015). The Empirical Foundations of Teledermatology: A Review of the Research Evidence. Telemed J E Health.

[6] Kontos E, Blake KD, Chou WS, Prestin A (2014). Predictors of eHealth usage: insights on the digital divide from the Health Information National Trends Survey 2012. J Med Internet Res.

[7] Carroll JK, Moorhead A, Bond R, LeBlanc WG, Petrella RJ, Fiscella K (2017). Who Uses Mobile Phone Health Apps and Does Use Matter? A Secondary Data Analytics Approach. J Med Internet Res.

[8] Graham AL, Amato MS (2019). Twelve Million Smokers Look Online for Smoking Cessation Help Annually: Health Information National Trends Survey Data, 2005-2017. Nicotine Tob Res.

[9] Jiang S, Street RL (2017). Factors Influencing Communication with Doctors via the Internet: A Cross-Sectional Analysis of 2014 HINTS Survey. Health Commun.

[10] McKoy K, Antoniotti NM, Armstrong A, Bashshur R, Bernard J, Bernstein D, Burdick A, Edison K, Goldyne M, Kovarik C, Krupinski EA, Kvedar J, Larkey J, Lee-Keltner I, Lipoff JB, Oh DH, Pak H, Seraly MP, Siegel D, Tejasvi T, Whited J (2016). Practice Guidelines for Teledermatology. Telemed J E Health.

[11] Mahmood A, Kedia S, Wyant DK, Ahn S, Bhuyan SS (2019). Use of mobile health applications for health-promoting behavior among individuals with chronic medical conditions. Digit Health.

[12] Connolly KK, Crosby ME (2014). Examining e-Health literacy and the digital divide in an underserved population in Hawai'i. Hawaii J Med Public Health.

[13] Cosco TD, Firth J, Vahia I, Sixsmith A, Torous J (2019). Mobilizing mHealth Data Collection in Older Adults: Challenges and Opportunities. JMIR Aging.

[14] Tennant B, Stellefson M, Dodd V, Chaney B, Chaney D, Paige S, Alber J (2015). eHealth literacy and Web 2.0 health information seeking behaviors among baby boomers and older adults. J Med Internet Res.

[15] Serrano KJ, Yu M, Riley WT, Patel V, Hughes P, Marchesini K, Atienza AA (2016). Willingness to Exchange Health Information via Mobile Devices: Findings From a Population-Based Survey. Ann Fam Med.

[16] Strekalova YA (2018). When Trust Is Not Enough: A Serial Mediation Model Explaining the Effect of Race Identity, eHealth Information Efficacy, and Information Behavior on Intention to Participate in Clinical Research. Health Educ Behav.

[17] AlGhamdi KM, Moussa NA (2012). Internet use by the public to search for health-related information. Int J Med Inform.

[18] Paige SR, Krieger JL, Stellefson ML (2017). The Influence of eHealth Literacy on Perceived Trust in Online Health Communication Channels and Sources. J Health Commun.

[19] Velsen LV, Tabak M, Hermens H (2017). Measuring patient trust in telemedicine services: Development of a survey instrument and its validation for an anticoagulation web-service. Int J Med Inform.

[20] Van Velsen L, Wildevuur S, Flierman I, Van Schooten B, Tabak M, Hermens H (2016). Trust in telemedicine portals for rehabilitation care: an exploratory focus group study with patients and healthcare professionals. BMC Med Inform Decis Mak.

[21] Almathami HKY, Win KT, Vlahu-Gjorgievska E (2020). Barriers and Facilitators That Influence Telemedicine-Based, Real-Time, Online Consultation at Patients' Homes: Systematic Literature Review. J Med Internet Res.

[22] Peracca S, Jackson G, Weinstock M, Oh D (2019). Implementation of Teledermatology: Theory and Practice. Curr Derm Rep.

[23] (2016). At Home. US Department of Veterans Affairs.

[24] Baranowski MLH, Balakrishnan V, Chen SC (2019). Patient Satisfaction with the Veteran's Administration Teledermatology Service. J Am Acad Dermatol.

